# Collaborative Genomics for Dystonia in Central and Eastern Europe: Successes Achieved, New Frontiers Ahead

**DOI:** 10.1002/mds.70300

**Published:** 2026-04-02

**Authors:** Robert Jech, Petra Havránková, Eugenia Tsoma, Lukáš Kunc, Tomáš Krajča, Ján Necpál, Olga Ulmanová, Aleksandra Tomic, Ivana Dzinovic, Irena Rektorová, Marek Baláž, Tereza Serranová, Stanislava Jaselska, Maria Giertlova, Kristina Kulcsarova, Alexandra Lackova, Denisa Harvanova, Miriam Ostrozovicova, Vladimir Han, GenDy Collaborators, Matej Škorvánek, Michael Zech

**Affiliations:** ^1^ Department of Neurology, 1st Faculty of Medicine and General University Hospital in Prague Charles University Prague Czech Republic; ^2^ Regional Clinical Center of Neurosurgery and Neurology, Department of Family Medicine and Outpatient Care Uzhhorod National University Uzhhorod Ukraine; ^3^ Department of Biomedical Informatics, Faculty of Biomedical Engineering Czech Technical University in Prague Kladno Czech Republic; ^4^ Department of Neurology Zvolen Hospital Zvolen Slovakia; ^5^ Department of Neurology Pavol Jozef Safarik University Kosice Slovakia; ^6^ Neurology Clinic University Clinical Center of Serbia Belgrade Serbia; ^7^ Institute of Human Genetics, School of Medicine and Health Technical University of Munich Munich Germany; ^8^ Institute of Neurogenomics, Helmholtz Zentrum München Munich Germany; ^9^ First Department of Neurology, St. Anne's University Hospital and Faculty of Medicine and CEITEC Masaryk University Brno Czech Republic; ^10^ Department of Pediatric Neurology Children's Faculty Hospital Kosice Slovakia; ^11^ Department of Pediatrics Faculty Hospital Banska Bystrica Slovakia; ^12^ Genetics Outpatient Clinic, Unilabs Kosice Slovakia; ^13^ Department of Neurology University Hospital of L. Pasteur Kosice Slovakia; ^14^ Department of Clinical Neurosciences Pavol Jozef Safarik University Kosice Slovakia; ^15^ Connected Tissue Bank, Pavol Jozef Safarik University Kosice Slovakia; ^16^ Institute for Advanced Study Technical University of Munich Garching Germany

**Keywords:** collaboration, data sharing, dystonia, genomics, underrepresented populations

The application and clinical utility of genomic analyses are well established in the field of movement disorders, where high‐throughput molecular tests have revolutionized the rate, speed, and precision of diagnosis.^1^ Nevertheless, there is a wide appreciation of inequitable access to genomic programs with maldistribution of enrollment into precision‐medicine studies for individuals living with a movement disorder in “underrepresented” geographical regions.^2^, ^3^, ^4^ Although successes in diversifying cohorts and overcoming diagnostic disparities have been shown for some forms of movement disorders, most notably for Parkinson's disease,^5^, ^6^ significant room for improvement of inclusion and investigation remains for other patient groups, such as individuals with dystonia. Lim and colleagues^7^ recently proposed a seminal definition of underrepresented populations in genomic research, highlighting patients from Eastern European countries as an important portion of underserved individuals who face barriers to systematic genetic characterization. The investigation of the impact of state‐of‐the‐art genomic testing approaches in such disadvantaged populations has often started to focus on more common conditions, exacerbating the issue of underrepresentation of patients with rare genetic dystonias from these regions.^7^ In this Viewpoint article, we aim to summarize our experiences in building a roadmap for narrowing disparities in genomic research among dystonia‐affected individuals from underrepresented European countries. Through the perspective of the “Mapping Genomic Diversity of Dystonia in Central and Eastern Europe” (GenDy) project, we report accomplishments and provide examples on a 10‐year timescale,^8^, ^9^, ^10^ fostering more equitable advantages from genomics for diverse patients with dystonia.

## Looking Backwards: Accomplishments of the Project

### Establishment of a Multicenter Cohort to Enhance Representativeness

**FIG. 1:**
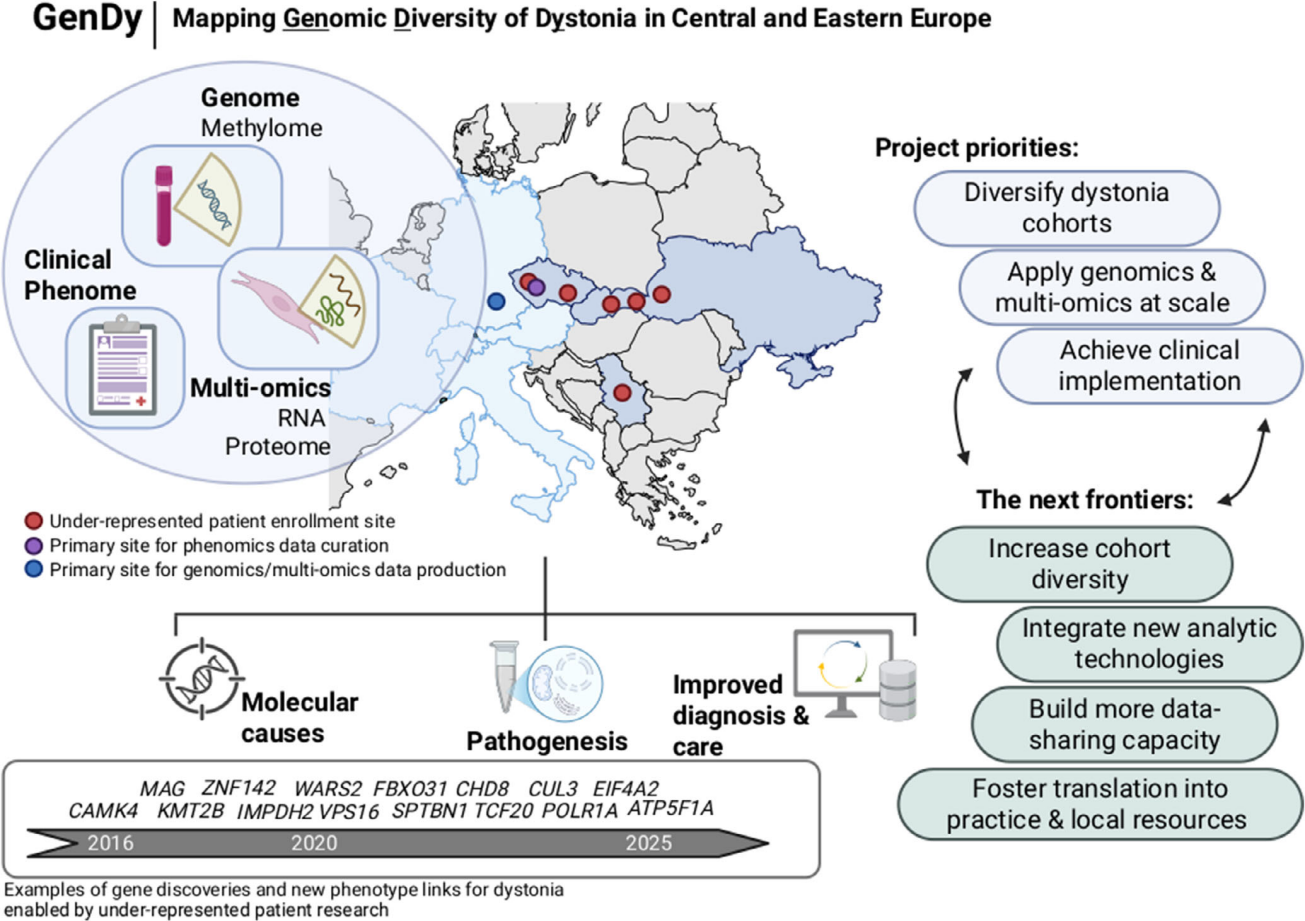
Overview of the Mapping Genomic Diversity of Dystonia (GenDy) project focused on genetic dystonias in Central and Eastern Europe (CEE). Participant enrollment sites, collected samples and information, project goals, and major gene discoveries on a 10‐year timescale are illustrated. The project has accelerated research in monogenic dystonia across different regions with traditionally underrepresented patient populations. The next planned steps are highlighted in green. BioRender was used to create (parts of) the figure (https://www.biorender.com). [Color figure can be viewed at wileyonlinelibrary.com]

Patients with dystonic diseases are typically geographically scattered, and the individual presentations are heterogeneous with limited options to predict specific etiologies based on clinical features.^11^ In many countries in Central and Eastern Europe (CEE), including Czech Republic, Slovakia, Serbia, and Ukraine, there has been a shortage of molecular testing services, with the unevenly dispersed genetics experts being concentrated in a few larger academic referral sites, hampering the widespread adoption of well‐defined diagnostic care protocols. In addition, while unbiased analysis approaches such as whole‐exome sequencing (WES) and whole‐genome sequencing (WGS) have been underutilized in routine settings for patients with dystonia from these countries, their molecular diagnostics has been mostly restricted to targeted screening for DYT‐*TOR1A* and DYT‐*THAP1*.^8^ Very little was known about the genetics of dystonia in CEE, presenting impediments to equitable outcomes from genomic studies and diagnosis‐informed clinical management for the affected families. Since 2015, the Charles University in Prague (Prague, Czech Republic) and the Technical University of Munich and Helmholtz Munich (Munich, Germany) partnered with additional centers in CEE (Czech Republic, Slovakia, Serbia, Ukraine; Fig. 1) as part of the GenDy project.^8^, ^9^, ^10^ This initiative aims to (1) achieve diversification of dystonia cohorts originating from CEE, (2) take large‐scale genomic and multi‐omic approaches for deepening our understanding of the molecular bases of dystonic syndromes, and (3) translate genomic‐medicine research in dystonia to the clinic (Fig. 1). Over the past decade, we have enrolled 1549 total research participants, including 961 index patients (mean age 31 years, range 1–81 years; 49% female), 56 affected family members, and 532 healthy relatives, following ethics review committee‐approved procedures and appropriate permissions. Addressing broader disparities by targeted outreach to multiple subspecialties, GenDy's recruitment has purposefully been inclusive.^8^, ^9^, ^10^ In addition to diverse isolated dystonic presentations, GenDy has had dystonia combined with other neurological, especially neurodevelopmental, signs as an important focus of investigation (64% of index patients); the enrolled individuals present a uniquely heterogeneous range of manifestations encompassing developmental delays, epilepsy, co‐occurring movement disorders, and other features.^8^, ^9^, ^10^ Multilevel phenotype information, including a standardized video collection, has been compiled and can be collaboratively (re)analyzed on a secure, web‐based platform (research electronic data capture, REDCap),^12^ facilitating downstream data interpretation and cross‐institutional evaluations.^13^ To enhance research productivity, a diversity of biospecimens suitable for genomics, multi‐omics, and functional studies have been ascertained, which are stored centrally at biobanks and can be made available to collaborating sites through committee review. The GenDy biorepository currently comprises 1549 different DNA samples, alongside a specialized collection of fibroblast lines from 100 affected individuals. Molecular characterization is ongoing, with 100 RNA sequencing and 60 proteomics datasets currently available,^10^, ^14^ and plans to complete these multi‐omic profiles for the remainder of the fibroblast cohort to facilitate integrated functional analyses.

## Lessons Learned: Genomic Discoveries, Improved Diagnosis, Insights into Pathogenesis, and Therapeutic Consequences

GenDy has so far applied genomic sequencing to 961 families using different analytical designs (250 trio analyses). The overall diagnostic yield from the main employed sequencing method—WES—within GenDy is 22%,^8^, ^10^ which accounts for 118 different rare monogenic diagnoses (most common diagnosis: *SGCE*). An analysis of all WES‐based genetic diagnoses across participating countries, detailed in Table S1, revealed a broad spectrum of causal genomic findings without evidence of significant regional enrichments. Instead, the genetic landscape appeared to be characterized by a few recurrently affected genes alongside a large number of ultra‐rare conditions identified only once,^10^ reflecting the rarity of these disorders rather than population‐specific structures or referral biases. However, regional differences in diagnostic yield were observed; for instance, the higher yield in the Slovakian subcohort likely reflects an enrichment for complex pediatric cases compared with regions with a higher proportion of isolated adult‐onset dystonia referrals. This underscores that the success of genomic screening in CEE is influenced by clinical stratification and the inclusion of early‐onset, more complex phenotypes. WES analysis of the first 186 GenDy index‐case participants (2015–2019) was instrumental in establishing a predictive scoring system,^8^ which today offers guidance to clinicians who seek to select dystonia‐affected subjects for genome‐wide diagnostic testing. GenDy has also led the way in discovering a number of novel disease genes for dystonia, which is a notable success since other large‐scale genomics programs have often failed to efficiently pursue the identification of undescribed gene candidates.^4^, ^15^ We presented molecular and clinical details on participants with previously unrecognized genotype–phenotype relationships associated with *CAMK4*,^16^ *IMPDH2*,^8^ *VPS16*,^17^ *ZNF142*,^18^ and several other genes (for a summary see Fig. 1). The efforts also uncovered founder mutations, such as a recurrent *VPS16* nonsense variant in individuals of Roma descent underlying generalized isolated dystonia.^19^ Aiming to resolve a wide spectrum of challenging rare dystonia cases, GenDy has recently incorporated WGS analyses for 167 participating families,^10^ identifying 25 previously missed diagnoses. The project has also started to generate valuable multi‐omic data that have added to the body of evidence required to confirm causality of variants in long‐term unresolved families.^10^ Beyond diagnostic utility, our experience in GenDy has provided relevant lessons about disease mechanisms and the roles of converging molecular pathways in dystonia.^11^ We now recognize that the pathogenesis of dystonic expressions involves substantial contributions from neurodevelopmental gene alterations, often impacting on key constrained cellular processes.^20^ Finally, the efforts resulted in a number of gene‐informed management changes, triggering improved clinical outcomes. For example, results have provided insights into therapy and/or surveillance measures in the context of variants in *ADCY5*, *ATM*, *CACNA1A*, *GCH1*, *GNAO1*, *NPC1*, *PAH*, *SPR*, *TTPA*, and others (for a summary see Zech et al.^10^).

## Benchmarking GenDy: Comparison with Other Global Initiatives

To contextualize the unique position of GenDy, it is instructive to compare its framework with major international benchmarks such as the Global Parkinson's Genetics Program (GP2)^6^ and the Dystonia Coalition.^21^ While GP2 has set a global precedent for large‐scale diversity in movement disorders, its primary focus remains on Parkinson's disease, leaving a significant gap in the systematic genomic mapping of dystonia within similar underrepresented regions. Compared with the Dystonia Coalition, both initiatives manage comprehensive genomic resources with hundreds of DNA samples each^21^; however, their cohort compositions and molecular analysis strategies diverge. The Dystonia Coalition repository predominantly includes isolated dystonia cases, with only a small fraction representing non‐focal forms.^21^ Consequently, this focus contributes to a relatively low diagnostic yield in WES of less than 10%, as reported in studies involving many samples of the coalition.^22^ In contrast, GenDy is more enriched for complex pediatric and generalized dystonia cases, which results in higher diagnostic yields and provides an important ground for WGS and multi‐omic applications^10^, ^14^ that are not yet available at scale within the Dystonia Coalition. Furthermore, while the coalition possesses more extensive collections of certain biosamples (eg, blood plasma),^21^ it lacks GenDy's specific geographic mandate for CEE. Despite differences in recruitment and methodology, all these projects—GP2, the Dystonia Coalition, and GenDy—pursue the common goals of fostering international collaboration and achieving cohort‐wide molecular characterization for future trial readiness,^6^, ^10^, ^21^ while facing shared challenges in maintaining sustainable funding and establishing systematic protocols for translating research output into daily practice.

## Progressing Forwards: The Next Frontiers

### Need to Further Increase Cohort Diversity

Despite the reported successes in the GenDy project, there is a critical role for continued research‐based molecular studies of dystonia‐affected individuals from CEE that increase the access to innovative testing approaches and bypass existing insurance restrictions. Notably, limited availability of sequencing and precision‐medicine initiatives coupled with budgetary constraints have been described as the most significant barriers to advancing genomic progress in underrepresented countries with less‐specialized public health services.^23^ An important challenge that we face is dealing with a large subset of the dystonia population that still lacks representativeness in genomic explorations. For example, we have not yet captured affected families from geographic sites where the local care teams experience shortages of movement disorder specialists and other knowledgeable practitioners, as in many isolated rural areas in CEE that fall outside academic medical center referrals. In addition, access challenges to tailored research for patients with dystonia in CEE include time constraints in primary care and perceived cultural inappropriateness (eg, in ethnic minority groups). Engagement of the Roma community, one of the largest European minority populations, in future dystonia genomic studies will be of pivotal importance. Expanded genomics‐first approaches will require more standardizations in return‐of‐results processes, follow‐up precision care, and patient engagement after unrevealing genomic testing.^24^, ^25^

### Harnessing New Technology and Bioinformatic Advances

Molecular testing approaches have rapidly evolved from specialized applications of WES in selected rare disease subjects to broader utilization of advanced technologies including WGS and complementary multi‐omic methods in larger groups of patients.^1^ RNA sequencing, proteomics, methylome analysis, and long‐read genome sequencing, supported by sophisticated bioinformatic pipelines, are emerging.^26^ There is a crucial need to put forth strategies to enhance equitable advantages from these newer techniques to geographically diverse patients, including those presenting with dystonia. Unless the utility of these tools over current conventional tests has been evaluated across multiple clinical settings and populations, their true potential remains untapped.^24^, ^27^ Defining the next best steps to achieve the objective of clinically useful implementation of first‐/or second‐line “multi‐omics diagnostics” for dystonic individuals from various geographical locations depends on carefully designed research studies. Unfortunately, investigators from CEE and other less‐privileged countries often face obstacles to obtain funding for multi‐omic investigations and pipeline development. In GenDy, we have begun to realize the value of overcoming the limitations of WES and WGS in undiagnosed families. We have recruited participants who remained without diagnostic‐grade findings after standard exome‐/genome‐wide screenings for investigation by dedicated multi‐omic workflows,^10^, ^28^ helping to meet the challenge of non‐interpretable or previously undetected disease‐causing variations. For example, the approach uncovered a distinct epigenetic signature for atypical *KMT2B*‐related dystonia in individuals of Czech origin.^29^ Variable expressivity in dystonic diseases has long been recognized, and methylation analysis may represent an effective strategy to decipher the biological basis of different population‐specific predispositions to this phenomenon. Multi‐omic tests require analysis‐appropriate tissues to yield relevant results.^1^ Thus, more biobanking of suitable biospecimens will need to be considered, addressing local logistical challenges in underrepresented regions.^25^

### Support of Data‐Sharing Practices

Another key strategy to increase diagnostic rates and cross‐cohort discoveries for patients with unrevealing genomic testing is data sharing.^30^ Case‐matching and networking have already played significant roles in elucidating the genetic causes in previously unresolved dystonia‐affected individuals from CEE.^8^, ^10^ Utilizing Matchmaker exchange platforms,^31^ we successfully contributed to global consortia aiming at unraveling new phenotype links for *CUL3*,^32^ *POLR1A*,^33^ and *SPTBN1*.^34^ Moreover, GenDy has identified compelling candidate variants in genes not yet associated with human disorders in another 63 families, awaiting establishment of causality through a globally‐shared evidence base.^31^ Recognizing the importance of capturing geographic diversity in community‐curated databases,^30^ we submitted >300 variant alleles with accompanying clinical significance information identified in GenDy participants to ClinVar.^35^ Combined with data from other laboratories, this may offer important insights into population‐specific disease prevalences in the future. We also continue to support network science by encouraging investigators to communicate directly with GenDy researchers regarding querying allele frequencies and candidate findings in our in‐house‐created genomic repository for dystonia cases from CEE. The need for more systematic data sharing, enabled by pan‐European initiatives,^36^ has become evident as we recognize the high number of unexplained disease presentations after molecular analysis. To address this, GenDy has started to work closely with national and international collaborative research infrastructures,^36^, ^37^ enhancing the contribution of underrepresented patient groups to harmonized projects.

### Accelerating Clinical Implementation in the Context of Local Requirements

The development of dedicated inclusion workflows and the training of local experts ensuring that patients achieve informed understanding of results would be needed to enable more widespread implementation of genomic analysis in the healthcare of affected individuals in this region. As part of GenDy, we adopted strategies to educate movement disorder specialists treating patients with monogenic dystonia in the communication of genomic findings and to increase their commitment to the ordering of molecular testing. However, much more systematic training of neurologists in CEE will be important to better address the existing barriers that limitations in knowledge about genomics have on accurate diagnoses for dystonic patients. Moreover, in order to make genomic investigations more accessible and affordable for patients with dystonia, specific economic analyses will likely be needed, providing arguments to policymakers and insurance companies for the benefits of precision medicine approaches.^3^, ^23^ Costs still represent a significant barrier to implementation of high‐throughout testing in resource‐limited countries,^7^ and therefore future studies comparing different testing regimes for individual indications will be useful for incorporating sustainable diagnostic solutions. To truly support equity, the integration of methods such as WES should become a priority for those dystonia‐affected individuals who are most likely to have an underlying monogenic condition,^38^ regardless of geographic location or limitations from insurance coverage.

## Conclusions and Strategic Roadmap

GenDy has fostered research capacity for various dystonic diseases in different countries in CEE with lower‐resourced primary care settings. We believe that the efforts have demonstrated the important value of broad access to genomic testing, shortening the diagnostic odysseys of affected families and bypassing bottlenecks of traditional genetic referrals. Looking forward, we hope that the real‐life impact of the project will result in wider region‐specific access to genomic testing, higher rates of precise diagnoses, and the availability of harmonized data for future collaborative initiatives aiming at advancing diagnostic equity and reducing between‐population care differences. To ensure the sustainability and impact of GenDy, we have begun to establish a strategic roadmap structured across 3‐, 5‐, and 10‐year intervals, with the core priorities of leveraging cohort expansion, new technology, and standardized data‐sharing practices. Within the next 3 years, our focus will be on a systematic expansion into additional countries (eg, Hungary) to reach a targeted recruitment rate of >300 new samples per year in GenDy. During this phase, we will increasingly harness Oxford Nanopore Technologies‐based long‐read sequencing^39^ and expand our multi‐omic pipeline,^10^, ^14^ while simultaneously preparing for the integration of our data into international repositories. By strengthening connections to the Dystonia Coalition and utilizing platforms like Matchmaker Exchange^40^ even more systematically, we will aim to facilitate the cross‐cohort analyses that are poised to accelerate the discovery of novel gene–disease associations. We expect that over the next 3–5 years, the integration of these technologies and additional reference populations will significantly improve the accuracy of genomic testing in the CEE region. Our 5‐year perspective involves transitioning toward full‐scale multi‐omics to bridge the gap between genetic discovery and genomics‐informed understanding of disease. The most challenging aspect of our 10‐year vision is the accelerated clinical implementation of these findings within the context of varied local requirements. Our goal is to see these genomic advances fully implemented into regional healthcare systems, establishing a sustainable genomics‐directed clinical trial network that ensures patients in underrepresented European regions have equal access to precision medicine.

## Author Roles

(1) Research Project: A. Conceptualization, B. Visualization, C. Data Curation; (2) Statistical Analysis: A. Design, B. Execution, C. Review and Critique; (3) Manuscript Preparation: A. Writing of the First Draft, B. Review and Editing, C. Approval of the Final Version.R.J.: 1A, 1C, 3A, 3B, 3C.

M.Z.: 1A, 1B, 1C, 3A, 3B, 3C.

M.S.: 1C, 3B, 3C.

P.H.: 1C, 3C.

E.T.: 1C, 3C.

L.K.: 1C, 3C.

T.K.: 1C, 3C.

J.N.: 1C, 3C.

O.U.: 1C, 3C.

A.T.: 1C, 3C.

I.D.: 1C, 3C.

I.R.: 1C, 3C.

M.B.: 1C, 3C.

T.S.: 1C, 3C.

S.J.: 1C, 3C.

M.G.: 1C, 3C.

K.K.: 1C, 3C.

A.L.: 1C, 3C.

D.H.: 1C, 3C.

M.O.: 1C, 3C.

V.H.: 1C, 3C.

GenDy Collaborators: 1C, 3C.

## Financial Disclosures of All Authors (for the Past 12 Months)

The authors report no disclosures.

## Supporting information


**Table S1.** Clinical characteristics of 961 index patients participating in GenDy and genes identified with causative variants per involved country.

## Data Availability

The data that support the findings of this study are available on request from the corresponding author. The data are not publicly available due to privacy or ethical restrictions.
