# Supplementary material for: Collaborative Genomics for Dystonia in Central and Eastern Europe: Successes Achieved, New Frontiers Ahead
Source: Mov Disord. 2026 Apr 2;41(7):1666–72. doi: 10.1002/mds.70300 (PMC13387963; doi:10.1002/mds.70300)
Supplement: Supplementary file 1 — Table S1. Clinical characteristics of 961 index patients participating in GenDy and genes identified with causative variants per involved country. [file MDS-41-1666-s001.docx]

**Supplementary Table 1** Clinical characteristics of 961 index patients participating in GenDy and genes identified with causative variants per involved country

| Country | Total  participants (index patients) | Isolated dystonia (%) | Early-onset dystonia (%) | Non-focal dystonia (%) | WES yield (%) | Genes identified with causative variants |
| --- | --- | --- | --- | --- | --- | --- |
| Czechia | 352 | 39.8% | 52.0% | 85.5% | 18.5% | *AARS1, ACTB, ADCK3, ADCY5, AFG3L, ANO3, ATM, ATP1A3, ATP7B, BRPF1, CAMK4, CAMTA1, CHD3, CHD8, CSDE1, EFTUD2, FOXG1, FRMD5, GNAL, GNAO1, GNB1, IFIH1, KCNA2, KIF5A, KMT2B, MICU1, NAV3, NKX2-1, PAH, PDE10A, PINK1, PNPT1, POLG, POLR1A, PRKCG, PRRT2, PSEN1, RAI1, SCGE, SGCE, SLC9A6, SOX6, SPG11, SPTAN1, SPTBN1, SYNE1, VPS16, WARS2, WDR45* |
| Serbia | 48 | 52.1% | 39.6% | 52.1% | 8.3% | *ANO3, VPS16, WARS2* |
| Slovakia | 424 | 26.7% | 72.9% | 90.1% | 26.7% | *ADAR, ADCY5, ALS2, ANK2, ARHGEF9, ATL1, ATM, ATP1A3, ATP2B2, ATP5F1A, AUTS2, C19orf12, CACNA1A, CD40LG, CHD4, CP, CTNNB1, CUL3, DJ1, DLG4, DNAJC6, EBF3, EIF4A2, ERCC4, FBXO31, FGF14, FOXG1, FTL, GABBR2, GCH1, GNAL, GNAO1, GRIA3, HECW2, HEXA, IMPDH2, IRF2BPL, KMT2B, MAG, MECP2, NEFL, NFIX, NGLY1, NPC1, PAK1, PCDH12, POLG, POLR3A, PPT1, PTS, RAI1, RHOBTB2, SATB1, SCN1A, SCO2, SETX, SGCE, SLC16A2, SLC6A3, SOX6, SPAST, SPG11, SPG7, SPR, TCF20, THAP1, TMEM240, TOR1A, TUBB4A, VPS16, WAC, WARS2, ZEB2, ZNF142, ZNF335* |
| Ukraine | 137 | 49.6% | 74.5% | 86.9% | 24.8% | *ADCK3, ADCY5, ATM, C19orf12, CACNA1A, COL4A1, FOXG1, GATA3, GCH1, GNAL, GNAO1, MBD5, PNKD, PURA, RHOBTB2, SGCE, SOX5, SUCLG1, THAP1, TOR1A, TTPA, VPS16, WARS2​​​​​​​​​​​​​​​​* |

WES, whole-exome sequencing
